# Incision and Drainage With Primary Fistulotomy of Perianal Abscess Is Safe and Effective in Neonates: A Long-Term Follow-Up Study

**DOI:** 10.3389/fped.2022.862317

**Published:** 2022-05-06

**Authors:** Wanbin Yin, Laian Li, Lin Su, Shuai Wang

**Affiliations:** ^1^Department of Anorectal Surgery, Affiliated Hospital of Jining Medical University, Jining, China; ^2^Department of Pediatric Surgery, Affiliated Hospital of Jining Medical University, Jining, China

**Keywords:** neonates, perianal abscess, incision and drainage, fistulotomy, fistula formation, abscess recurrence, long-term, follow-up

## Abstract

**Objective:**

Perianal abscess (PA) in neonates is poorly understood, and its management remains controversial. The aim of this study was to compare incision and drainage (ID) with or without primary fistulotomy in the management of neonatal first-time PA.

**Methods:**

A retrospective comparative study was conducted for neonates with first-time PA treated with incision and drainage with primary fistulotomy (IDF) vs. ID between 2008 and 2017.

**Results:**

In total, 138 patients (137 boys and 1 girl) were identified; 65 in the IDF group and 73 in the ID group. The median follow-up was 6.5 years (range 4–13 years). Baseline characteristics were similar between the 2 groups. The cure rate in the IDF group (98.5%, 64/65) was significantly higher than that in the ID group (80.8%, 59/73; *p* = 0.001). The rate of fistula formation in the IDF group (1.5%, 1/65) was significantly lower than that in the ID group (13.7%, 10/73; *p* = 0.01). The rate of abscess recurrence was not statistically different (*p* = 0.12), even though the IDF group (0%, 0/65) seemed to have a better outcome than the ID group (5.5%, 4/73). No fecal incontinence was observed in any of our patients.

**Conclusions:**

First-time PA in neonates can be treated safely and effectively by the IDF or by ID alone. The former may be advantageous over the latter in terms of the rate of cure and fistula formation.

## Introduction

A perianal abscess (PA) in neonates is a relatively common disease, but it is poorly understood. It is usually considered a trivial disease by physicians. Nevertheless, the discovery of perianal swelling in a neonate can be a source of anxiety for parents. Parents' excessive worry and physicians' insufficient experience make the management of neonatal PA very challenging. However, only rare literature is devoted to it. Therefore, the treatment is usually based on the studies of PA in infants and children.

The management of PA in infants and children is controversial. It has traditionally been treated surgically by incision and drainage (ID) (1–11). However, in the past two decades, some studies have shown that the conservative management appears to be effective in selected cases (12–23). On the other hand, the other studies have recommended that the surgical procedure should involve a careful identification for the fistula and treatment of that by fistulotomy (24–32). Thus, at present, the optimal management has not yet been established.

At our center, PA in infants can be treated either in the department of anorectal surgery or in the department of pediatric surgery. Some infants experienced fistula formation after ID, and were transferred to the department of anorectal surgery. Is ID enough? Is incision and drainage with primary fistulotomy (IDF) better than ID? These ideas prompted us to complete this long-term comparative study.

To the best of our knowledge, no published study in PubMed has directly compared the outcomes of IDF vs. ID for neonatal PA. This study aimed to compare the efficacy and safety of IDF and ID for PA in neonates.

## Materials and Methods

A retrospective case note review was carried out for all consecutive neonates with PA at a single tertiary center between January 2008 and December 2017. At our center, all neonates admitted to the department of anorectal surgery underwent IDF, while all neonates admitted to the department of pediatric surgery underwent ID. Patients with PA undergoing IDF were assigned to the IDF group, and those undergoing ID were assigned to the ID group. Although group allocation was not randomized, it was naturally formed according to the department in which the patients were hospitalized. Neonates with PA received intravenous antibiotics routinely (Ceftazidime in the IDF group and Cefathiamidine in the ID group) for 3–5 days.

The present study was approved by the Affiliated Hospital of Jining Medical University Institutional Review Board. Written informed consent was obtained from all parents.

### Diagnostic Criteria

Perianal abscess was diagnosed by the presence of a firm or fluctuant tender mass close to the anus. Fistula-in-ano (FIA) was diagnosed by the presence of a hole with or without pus drainage at the site of the anus, persisting more than 3 weeks postoperatively. A recurrence of PA was defined as an abscess developed at the original location again after wound healing. New-onset PA was defined as a new abscess that developed at other locations.

### Inclusion and Exclusion Criteria

Inclusion criteria were neonatal patients with first-time PA who presented to our center during the first 28 days after birth and underwent IDF or ID. Exclusion criteria were patients whose age at symptom onset is younger than 28 days but the age at admission was older than 28 days, inpatients discharged without surgery due to parental refusal, patients who had undergone prior surgical treatment at other centers, or patients lost to follow-up.

### Incision and Drainage

A small incision was made through the dome of the abscess and pockets within the cavity were delicately broken by gentle exploration with a hemostatic forceps under local anesthesia.

### Incision and Drainage With Primary Fistulotomy

Subsequent fistulotomy was performed right after ID with the patient in the left lateral decubitus position under conscious sedation and local anesthesia. The most important step was to identify the internal opening. A fine probe was gently introduced through the abscess cavity to the affected anal crypt, then the fistula tract was unroofed and laid open with diathermy. If the internal opening could not be probed, then the corresponding internal opening, which was in the same location as the center of the abscess was laid open. All of the fistulas were low-type.

### Follow-Up

Short-term follow-up was conducted by outpatient reviews within 3 weeks of discharge. Given the coronavirus disease 2019 (COVID-19) pandemic, long-term follow-up data were mainly obtained *via* telephone interview, supplemented by outpatient review and a home visit. As we all know, a high percentage of completed follow-up is essential for long-term outcomes and is necessary to minimize any bias that might result from failure to contact patients. Therefore, we made every effort to make contact with each parent.

### Data Collection

Collected data included baseline characteristics, length of follow-up, fistula formation, recurrence of abscess, new-onset abscess, and fecal incontinence.

### Statistical Analysis

Quantitative variables were summarized as mean ± standard deviation (SD) or median with interquartile range (IQR). Categorical variables were summarized as percentages. Comparisons between 2 groups were made by using the 2-tailed *t*-test or Mann–Whitney *U*-test for continuous variables. The chi-square test and Fisher's exact test were used for comparing categorical variables. Differences were considered statistically significant at *p* < 0.05. Data were analyzed using the SPSS software, version 18.0.

## Results

**Figure 1:**
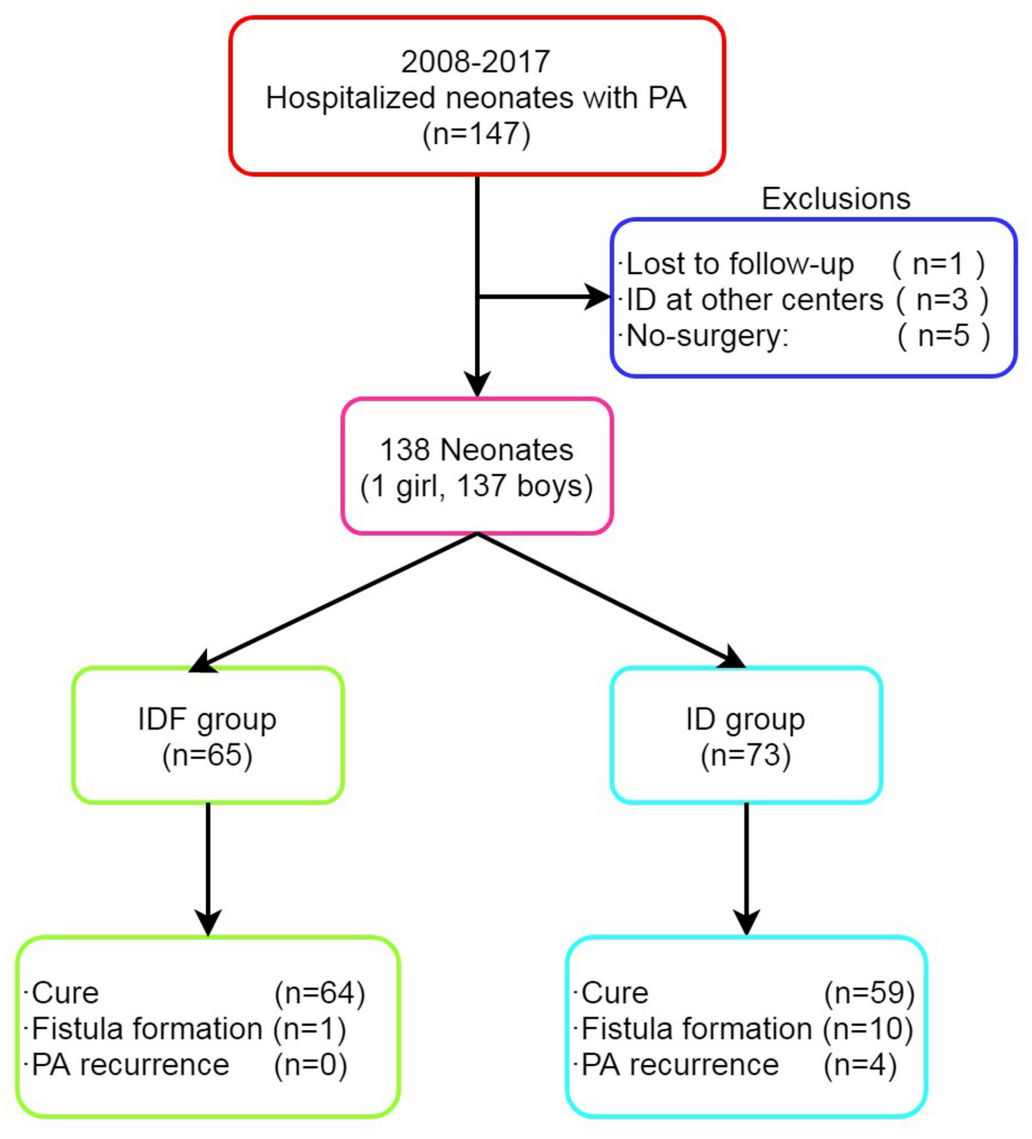
Enrollment, treatment, and long-term outcomes of patients with PA. PA, perianal abscess; ID, incision and drainage; IDF, incision and drainage with primary fistulotomy.

A total of 147 hospitalized neonates with PA were identified from January 2008 to December 2017 (Figure 1). Of the 9 excluded patients, 5 were excluded because parents refused surgery. Of the 138 included patients, 65 (47.1%) underwent IDF and 73 (52.9%) underwent ID. Follow-up data were available for all but one who had been excluded according to exclusion criteria. The median follow-up was 6.5 years (range 4–13 years).

Patient and abscess characteristics are summarized in Table 1. The 2 groups were similar in respect of gender, age of onset, duration of symptoms, age at admission, number of the abscess, location of the abscess, and length of stay. Of note, inpatient costs were higher in the IDF group vs. the ID group (3,774.0 vs. 2,416.0 Yuan, *p* = 0.000). Healing time (the time needed for the complete epithelialization of the wound bed) was about 3 and 2 weeks for the IDF group and ID group, respectively.

**Table 1:** Clinical characteristics of neonates with PA.

**Characteristics**	**IDF (*n* = 65)**	**ID (*n* = 73)**	***P***
Male sex, *n* (%)	65 (100)	72 (98.63)	1.00
Age of onset, mean ± SD, d	18.62 ± 4.28	18.73 ± 4.00	0.87
Duration of symptoms, median (IQR), d	4.00 (2.00–5.50)	3.00 (2.00–5.00)	0.77
Age at admission, mean ± SD, d	22.71 ± 3.49	22.36 ± 4.51	0.61
Number of the abscess, *n* (%)			0.13
1	58 (89.23)	70 (95.89)	
2	6 (9.22)	3 (4.11)	
3	1 (1.54)	0 (0)	
Locations of the abscesses			0.76
3-o'clock, *n* (%)	21 (28.77)	25 (32.89)	
9-o'clock, *n* (%)	35 (47.95)	32 (42.11)	
Other locations	17 (23.29)	19 (25.00)	
Length of stay, median (IQR), d	6.00 (5.00–7.75)	6.00 (5.00–8.00)	0.37
Inpatient costs, median (IQR), *yuan RMB*	3,774 (3,497–4,007)	2,416 (1,884–3,245)	0.000

*PA, perianal abscess; ID, incision and drainage; IDF, incision and drainage with primary fistulotomy; SD, standard deviation; IQR, interquartile range; RMB, renminbi, the currency of China*.

**Figure 2:**
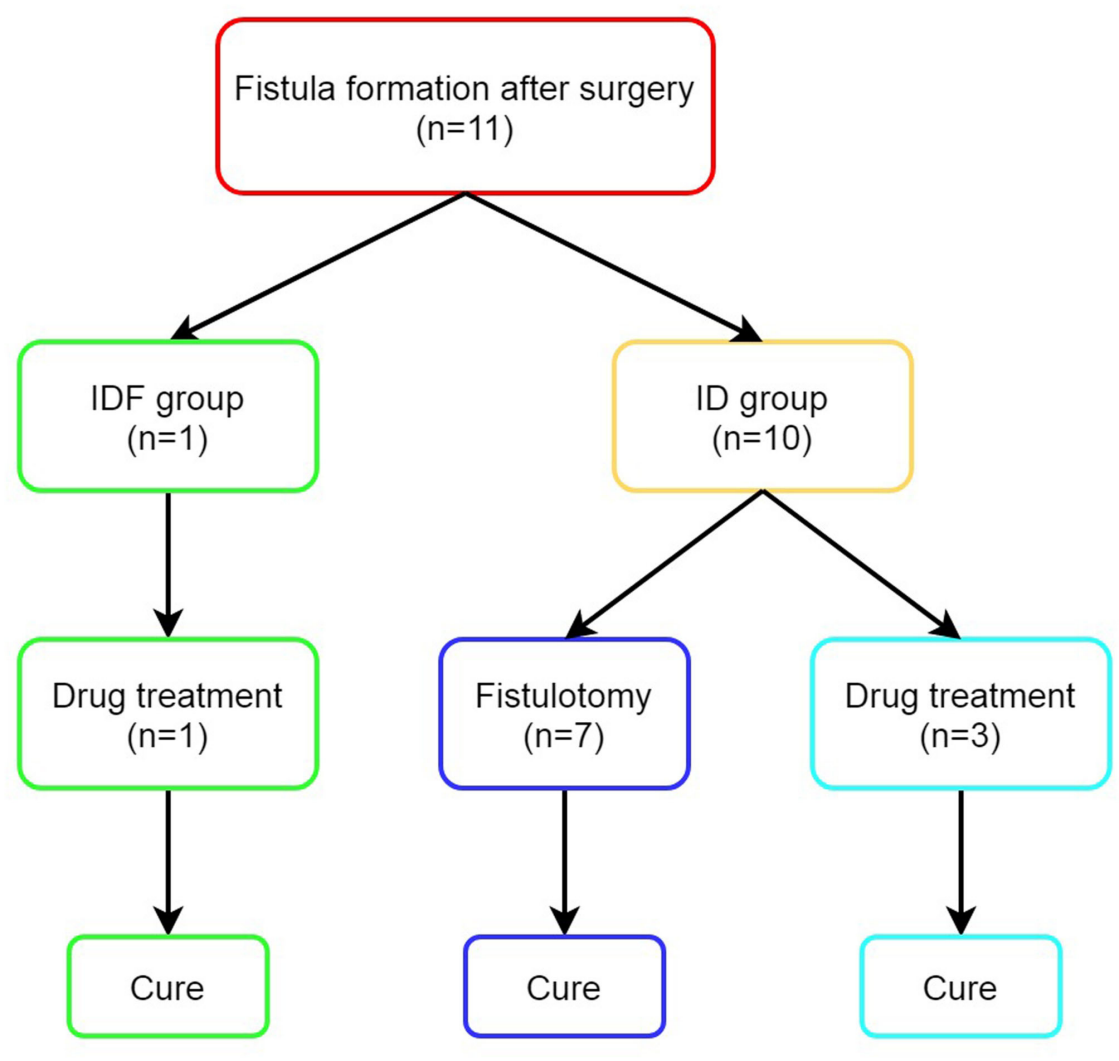
Outcomes of patients with fistula formation after surgery. ID, incision and drainage; IDF, incision and drainage with primary fistulotomy.

A comparison of long-term outcomes between the 2 groups is shown in Table 2. The median follow-up for patients undergoing IDF and ID was 5.00 years (IQR, 4.00–7.50) and 7.00 years (IQR, 5.50–9.00), respectively (*p* = 0.000). Patients in the IDF group had a higher cure rate (98.46 vs. 80.82%, *p* = 0.001) and a lower fistula formation rate (1.54 vs. 13.70%, *p* = 0.01) than the ID group. After surgery, 11 patients developed FIA (Figure 2). Of the 3 patients developing FIA after ID, 2 patients were cured after more than 2 years of plaster and Chinese herbal preparations ointment (ingredients unknown), respectively. The third patient was cured with an ointment (ingredients and duration unknown).

**Table 2:** Long-term outcomes of IDF vs. ID.

**Long-term outcomes**	**IDF**	**ID**	***P***
	**(*n* = 65)**	**(*n =* 73)**	
Follow-up time, median (IQR), y	5.00 (4.00–7.50)	7.00 (5.50–9.00)	0.000
Cure, *n* (%)	64 (98.46)	59 (80.82)	0.001
Fistula formation, *n* (%)	1 (1.54)	10 (13.70)	0.01
PA recurrence, *n* (%)	0 (0)	4 (5.48)	0.12

*ID, incision and drainage; IDF, incision and drainage with primary fistulotomy; IQR, interquartile range; PA, perianal abscess*.

None of the patients in the IDF group had a recurrence, while 4 patients in the ID group experienced recurrence (Table 3). Patients undergoing IDF seemed to have a lower abscess recurrence rate (0 vs. 5.48%; *p* = 0.12), albeit statistical significance was not achieved.

**Table 3:** Characteristics and course of recurrent patients after initial surgery^a^.

**Case**	**Time to recurrence**	**Location**	**Treatment**	**Outcome**
1	15 d	Right front	Re-ID	Cure
2	18 d	Right	IDF	Cure
3	78 d	Right front	Re-ID	Fistula formation^b^
4	20 m	Right front	IDF	Cure

*ID, incision and drainage; IDF, incision and drainage with primary fistulotomy; d, day; m, month*.

a *There was no recurrence among patients undergoing IDF*.

b *The patient was cured after subsequent IDF*.

It is noteworthy that 2 (3.08%) patients developed new abscesses at the contralateral side of the anus in the IDF group, compared with 7 (9.59%) patients in the ID group (Table 4). However, the difference between the 2 groups is not statistically significant (*p* = 0.17).

**Table 4:** Characteristics and course of patients with new-onset abscess after initial surgery.

**Case**	**Initial surgery**	**Time to new-onset**	**Location**	**Treatment**	**Outcome**
1	IDF	36 d	Left	Re-IDF	Cure
2	IDF	1 y	Left	Re-IDF	Cure
3	ID	2 d	Left	Re-ID	Recurrence^a^
4	ID	3 d	Right	Re-ID	Cure
5	ID	4 d	Left	IDF	Cure
6	ID	15 d	Right	CHM	Cure
7	ID	17 d	Right	IDF	Cure
8	ID	Not clear	Right	Plaster	Fistula formation^b^
9	ID	Not clear	Left	Re-ID	Cure

*ID, incision and drainage; IDF, incision and drainage with primary fistulotomy; CHM, Chinese herbal medicine; d, day; y, year*.

a *The patient was cured by conservative treatment*.

b *The patient was cured after 2 years of plaster*.

No fecal incontinence was confirmed in any patients in our study. A 10-year-old boy (undergoing IDF in 2010) with mental illness was found to have stools in his underwear sometimes. It is not sure whether it was caused by surgery or mental illness.

## Discussion

Our long-term study showed that IDF was associated with a higher rate of cure and a lower rate of fistula formation compared with ID. In addition, patients in the IDF group seemed to have a lower rate of abscess recurrence and new-onset abscess, albeit statistical significance was not achieved.

In our study, the cure rate in the IDF group and ID group was 98.46 and 80.82%, respectively. That was similar to a previous report (29) that cure occurred in 61/66 (92%) in whom a fistula was identified and treated by fistulotomy at the initial operation, compared with 19/25 (76%) in whom a fistula was not identified (ID alone). The high cure rate is attributable to careful identification and laying opening of the coexisting fistula. The rate of fistula (between the PA and the anal crypt) identified at the time of primary drainage varies widely in different studies. The percentage of communication between the PA and the internal (anal) opening was <20% (1, 4, 6, 7, 11) or more than 60% (25, 27, 29–32). Our results (approximately 80%) support the latter.

In the IDF group, the fistula formation rate was 1.54% (1/65) and there were no recurrences after surgery. Our findings are supported by several studies. There were no recurrences (26, 27, 31) or the recurrence rate was <15% (29, 30, 32) in patients who underwent fistulotomy at the time of ID. These findings are in excellent agreement with ours. Given this, we recommend that ID and laying open of the coexisting fistulous tract be performed for PA in neonates.

Surprisingly, in the ID group, the rate of fistula formation and recurrence was 13.7 (10/73) and 5.48% (4/73), respectively. These results are better than we expected. Our study with a large sample size confirms that neonates may have a lower rate of fistula formation after ID than infants and children. We believe that our results are valuable to the clinician. In the literature, the rate of fistula formation and recurrence was 10.5 (4/38) and 7.9% (3/38) (5), 6 (2/33) and 12% (4/33) (7), 20 (20/100) and 27 (27/100) (8), 30 (15/50) and 4% (2/50) (9), 3.8 (1/26) and 3.8% (1/26) (23), and 28 (8/29) and 7% (2/29) (24), respectively. It is well-known that fistula formation and abscess recurrence are the two major challenges for PA therapy. Unfortunately, some studies did not distinguish between them (1–3, 11, 14, 18, 29–34). Of those studies, the rate of fistula formation or recurrence after ID varies widely, which was <20% in 2 studies (2, 3), between 20 and 50% in 7 studies (1, 18, 29–32, 34), and more than 50% in 2 studies (14, 33). Altogether, previous studies differed substantially regarding the rate of fistula formation or recurrence after ID. There are several potential reasons for this difference. First, some reports did not distinguish between fistula formation and abscess recurrence. Since fistula formation and abscess recurrence are different, we call for distinguishing them in future studies. Second, the indications for ID varied in the literature. Some surgeons argue that the abscess should be incised as soon as possible, while other surgeons recommend ID should be reserved for patients with failed conservative management, large abscess, or systemic signs of infection. Third, surgical techniques may differ in detail. Fourth, the sample size in some studies was small. Thus, these results should be viewed with caution.

Remarkably, in the present study, 6.5% (9/138) of patients developed new abscesses on the contralateral side of the anus. Several studies have previously reported a similar phenomenon (16, 21, 25, 35, 36), but the reason was unknown.

Some studies show that PA is a self-limited entity. In our study, 4 patients who were excluded, experienced spontaneous drainage, and their parents refused surgery. Notably, 3 patients among them were cured without surgery. Similar results have been reported in other studies (18, 22). However, two other studies have shown different results (10, 12). We believe that there is a possibility of a self-resolution of abscesses. In future studies, we will further investigate the safety and effectiveness of non-operative therapy for PA in neonates who spontaneously drained.

There are several strengths to our study. First, the subjects in this study were neonates with PA, excluding infants and children. It is well-known that different stages of life have different pathophysiological features. PA in neonates, infants, and children may have different clinical characteristics. Limiting the age to newborns helped reduce the potential confounding effects of age and made the conclusions more reliable. Second, to the best of our knowledge, this is the first study directly comparing the outcomes of IDF vs. ID for neonatal PA. Third, our sample size (138 neonates) was very large, which improved the power of the analysis and reduced the bias to a certain extent. Fourth, follow-up data were available for all but one who had been excluded according to exclusion criteria. This is a long-term study with a median follow-up time of 6.5 years. Although various difficulties were encountered, with the aid of the public security bureau, all but one patient were successfully contacted due to the ingenuity, perseverance, and intense effort of our team. Finally, our study included detailed characteristics and course of patients with fistula formation, abscess recurrence, and new-onset abscess after initial surgery, which have not been reported previously.

Of course, some limitations in our study need to be recognized. First, it is a single-center retrospective study, potentially limiting the generalizability of our results. Second, long-term follow-up data were mainly obtained by telephone interviews instead of outpatient reviews. Considering the COVID-19 pandemic, this may be a reasonable alternative method. Third, patients' assignment was not randomized. However, we could be rather certain that it was not assigned by disease severity. At our center, PA in neonates can be treated at the department of anorectal surgery or the department of pediatric surgery. Why patients are admitted to a ward or to another depends on the cognition of the parents, not the severity of the disease. In addition, because of changes in the electronic medical records system, much data were not recorded consecutively, such as body temperature, abscess size, body mass index, white blood cell count, C-reactive protein, procalcitonin, and pus cultures. However, our results showed that important clinical characteristics that might influence the outcome were considerable between the 2 groups (Table 1). Thus, there was no inclusion bias. Fourth, not all the surgeries were performed by the same surgeon. Nevertheless, all of those were performed by experienced surgeons using the standardized technique in each group. Finally, the children are too young to cooperate, so anorectal manometry was not performed, which should be performed in the near future.

Unfortunately, PA in neonates and infants is generally considered a trivial condition. However, there are considerable unresolved questions surrounding it. Long-term prospective studies will be necessary to determine the optimal treatment.

## Conclusion

Our long-term follow-up study demonstrated that IDF and ID are both safe and effective treatments for PA in neonates. IDF is associated with a higher rate of cure and a lower rate of fistula formation compared with ID. Our findings support the value of careful identification and laying opening of the coexisting fistula when ID is performed.

## Data Availability Statement

The original contributions presented in the study are included in the article/supplementary material, further inquiries can be directed to the corresponding author/s.

## Ethics Statement

The studies involving human participants were reviewed and approved by Affiliated Hospital of Jining Medical University. Written informed consent to participate in this study was provided by the participants' legal guardian/next of kin.

## Author Contributions

WY: study design, data collection, and drafting of the manuscript. LL: data collection and analysis, article revision, and quality control. LS: data collection, statistical analysis, and article revision. SW: study design, data collection, article revision, and overall responsibility for the article. All authors read and approved the final manuscript.

## Conflict of Interest

The authors declare that the research was conducted in the absence of any commercial or financial relationships that could be construed as a potential conflict of interest.

## Publisher's Note

All claims expressed in this article are solely those of the authors and do not necessarily represent those of their affiliated organizations, or those of the publisher, the editors and the reviewers. Any product that may be evaluated in this article, or claim that may be made by its manufacturer, is not guaranteed or endorsed by the publisher.
